# Behavioral approach to autism spectrum disorder: quality versus quantity in interventions

**DOI:** 10.1016/j.jped.2025.101451

**Published:** 2025-10-07

**Authors:** Jaime Lin, Júlio César Claudino dos Santos, Cinara Ludvig Gonçalves

**Affiliations:** a Universidade do Extremo Sul Catarinense (UNESC), Criciúma, SC, Brazil; b Universidade Federal Fluminense (UFF), Niterói, RJ, Brazil

**Keywords:** Autism spectrum disorder, Behavioral interventions, Evidence-based practice, Quality of care, Professional training

## Abstract

**Objective:**

To discuss the importance of balancing quality versus quantity in behavioral interventions for individuals with Autism Spectrum Disorder (ASD), highlighting evidence-based approaches and the role of therapist training.

**Data sources:**

Narrative review of the literature examining evidence-based behavioral approaches for ASD, the tension between intervention intensity and quality, factors influencing individualized treatment planning, and the importance of professional qualification.

**Summary of findings:**

Evidence indicates that more hours of therapy do not necessarily result in better outcomes, with studies showing no consistent dose–response relationship. Individual learning rates, comorbid conditions, and quality of implementation significantly influence results. High-quality, individualized planning, consistent execution, family engagement, and well-trained professionals are essential. Lack of regulation and standardized training, particularly in contexts without professional certification systems, poses challenges to delivering effective, evidence-based care.

**Conclusion:**

Behavioral interventions for ASD must prioritize quality over quantity, ensuring evidence-based, individualized, and well-supervised treatment plans delivered by qualified professionals to achieve meaningful outcomes.

## Introduction

Autism Spectrum Disorder (ASD) is a neurodevelopmental condition characterized by difficulties in social communication and the presence of restricted and repetitive behaviors, with varying degrees of severity. Clinical presentations are heterogeneous and may range from individuals with intellectual disability and limited communication to those with above-average intellectual functioning but marked difficulties in social interaction.^1^

This clinical heterogeneity, which makes ASD an inherently complex condition, arises from factors related to neurodevelopment, such as age and intelligence quotient (IQ); environmental factors, spanning from perinatal risks to the availability of appropriate therapeutic interventions; and the presence of comorbidities such as epilepsy, sleep disturbances, and associated psychiatric disorders.^2^

From an epidemiological perspective, in the United States, the Autism and Developmental Disabilities Monitoring (ADDM) Network, linked to the Centers for Disease Control and Prevention (CDC), has published biennial prevalence estimates for ASD in 8-year-old children since 2000. A marked increase has been observed: from 1 in every 150 children to 1 in every 31 in the most recent report, published in 2025.^3^ Globally, systematic reviews of prevalence studies estimate that approximately 1% of the pediatric population has an ASD diagnosis.^4^ Based on these data, it is estimated that over two million children in Brazil are on the spectrum.

Multiple factors may explain the rising prevalence of ASD, including changes in diagnostic criteria, greater public awareness, expanded access to screening and diagnostic services, the development of specialized early behavioral intervention programs, individualized educational plans, and the possibility of a real increase in cases due to genetic and environmental interactions.^5^

However, while earlier and broader access to diagnosis represents a significant advance, it also carries the responsibility to ensure access to effective, evidence-based interventions. Unfortunately, the rise in incidence and public awareness has been accompanied by a proliferation of treatment options with limited or no scientific support.

In particular, within the field of behavioral interventions, a critical question emerges: how can the authors balance intensity (number of hours) with the technical and clinical quality of the practices used? Although intensive programs are often recommended, not all extensive interventions yield meaningful results if they are not structured, personalized, and evidence-based. This review aims to provide pediatricians with a critical and practical perspective on this tension between “quantity” and “quality” in behavioral approaches to ASD, supporting them in recommending effective, evidence-based therapeutic options.

## Behavioral approach to autism spectrum disorder: evidence-based interventions

The primary reason for encouraging early identification and diagnosis of ASD is that interventions begun before age 3, when the brain exhibits greater plasticity, are more likely to have long-term effectiveness. The use of evidence-based early intervention services that promote sequential skill development and positive parent–child interactions is associated with better outcomes.^6^

There is a broad consensus among researchers, clinicians, advocates, and policymakers that individuals on the autism spectrum should receive evidence-based interventions. An “evidence-based” intervention is one whose beneficial effects have been objectively demonstrated through rigorous scientific research, rather than relying on individual case reports or subjective impressions.^7^

Evidence-based behavioral approaches can be defined as interventions recognized as effective and safe for treating people with ASD, whose efficacy has been demonstrated in multiple studies published by independent research teams.^8^ Additionally, it is essential to consider the knowledge and experience of healthcare professionals in applying scientific evidence to each patient's clinical context, as well as the preferences and values of the family. Actively involving patients and caregivers in treatment decisions is fundamental, respecting their personal expectations, cultural values, and goals.

Interventions for children with ASD can be delivered through educational practices, developmental therapies, and behavioral interventions. Their aims include minimizing core deficits and associated impairments, maximizing functional independence, and preventing problem behaviors that interfere with adaptive skills. These interventions can be implemented in diverse settings by a variety of professionals, following defined curricula or guidelines.^9^

Grounded in learning principles, behavioral therapies use specific techniques, such as reinforcement, to promote positive behaviors and reduce those that may have negative consequences for the individual or others in their environment.^10^

Broadly, behavioral therapies for ASD can be divided into two major categories. Focused intervention practices are designed to address a single skill or goal for a child with autism, aiming for clearly defined outcomes. Teachers, clinicians, or other professionals select and apply operationally defined practices (e.g., prompting, reinforcement, time delay) in individualized instructional contexts.^8^ In contrast, comprehensive program models consist of an integrated set of practices designed to produce a broad impact on learning and development by targeting ASD’s core deficits. These programs are organized around a conceptual framework, address a wide range of goals, and are typically implemented for a substantial number of hours per week over one or more years.^8^

The most recent review of intervention practices for ASD, conducted by the National Clearinghouse on Autism Evidence and Practice (NCAEP) and published in April 2020, analyzed research on focal interventions in autism and identified 28 Evidence-Based Practices. Of these, 23 are grounded in Applied Behavior Analysis (ABA), while five others are: 1) Exercise and Movement; 2) Music-Mediated Intervention; 3) Technology-Aided Instruction and Intervention; 4) Cognitive Behavioral Intervention; and 5) Ayres Sensory Integration, each with its own theoretical framework.^11^

Applied Behavior Analysis (ABA) is a scientific approach focused on understanding and modifying human behavior through the systematic application of behavioral principles. It relies on operant and respondent conditioning to identify and change environmental factors that influence behavior. ABA involves analyzing functional relationships between behaviors and the environment to develop socially acceptable alternatives to problematic behaviors, often using strategies such as differential reinforcement [12]. Table 1 presents the complete list of the 28 evidence-based practices for ASD treatment, according to the NCAEP review.

**Table 1:** Evidence-based practices for the treatment of ASD according to the NCAEP review.

**Evidence-Based Practice**	**Definition**
Antecedent-based interventions (ABI)	Organization of events or circumstances that precede an activity or demand, in order to increase the occurrence of behaviors or lead to a reduction in challenging/inappropriate behaviors.
Alternative and augmentative communication (AAC)	Interventions using and/or teaching the use of a nonverbal/vocal communication system, including assisted and unaided communication systems. Unaided communication systems do not use any material or technology (e.g., sign language and gestures). Assisted communication systems include low-tech systems (e.g., object/picture exchange or pointing to letters) and extend to high-tech speech-generating devices and applications that enable other devices (i.e., phones, tablets) to serve as Speech Generating Devices (SGDs).
Behavioral Momentum Intervention (BMI)	It is a strategy in which the presentation of the task is modified so that those requiring less laborious responses (i.e., high-probability response sequences) occur before those requiring more difficult responses (i.e., low-probability response sequences). This is done so that students receive reinforcement earlier and are more likely to remain engaged and persist with the more challenging tasks or requests that follow.
Cognitive behavior/instructional strategies (CBIS)	Students are taught to examine their own thoughts and emotions and then use step-by-step strategies to change their thinking, behavior, and self-awareness. These interventions can be used with students who exhibit problematic behaviors related to specific emotions or feelings, such as anger or anxiety (e.g., cognitive behavioral therapy). These interventions can also be used to support students in acquiring social and academic skills through explicit instruction on learning strategies. CBIS interventions are often used in conjunction with other evidence-based practices, including modeling, visual supports, prompts, reinforcement, social narratives, instruction, and peer-based and parent-implemented interventions.
Differential reinforcement of alternative, incompatible, or other behavior (DR)	It is a systematic process that increases desirable behavior or the absence of undesirable behavior by providing reinforcing consequences for the demonstration/non-demonstration of certain behavior. Undesirable behaviors are those that interfere with the development, relationships, and health of the person being served (e.g., disengagement, tantrums, aggression, self-harm). Differential reinforcement is often used with other evidence-based practices, such as the use of cues to encourage more desirable behaviors or behaviors that are incompatible with the interfering behavior.
Direct Instruction (DI)	A systematic approach to teaching using a package of sequenced instructions, with scripts or lessons. It emphasizes teacher-student dialogue through choral and independent responses that enable systematic and explicit correction of errors to promote learning and generalization.
Discrete Trial Training (DTT)	Discrete Trial Teaching (DTT) is an individual instructional approach (usually) used to teach skills in a planned, controlled, and systematic manner. DTT is characterized by repeated or grouped trials that have a defined beginning and end. In DTT, the use of antecedents and consequences is carefully planned and implemented. Instruction begins when the practitioner presents a clear direction or stimulus, which elicits a target behavior. Praise and/or tangible rewards are used to reinforce the desired skills or behaviors.
Exercise and movement (EXM)	Interventions that use physical effort, specific motor skills/conscious movement techniques to target a variety of behavioral skills. Exercise can be used as a preceding activity to improve performance on a task or behavior or to increase physical fitness and motor skills. Movement activities may include sports/recreational activities, martial arts, yoga, or other mind/body practices focusing on specific groups of motor skills and techniques.
Extinction (EXT)	It consists of removing reinforcing consequences of challenging behavior in order to reduce the future occurrence of that behavior. The extinction procedure depends on accurately identifying the function of the behavior and the consequences that may be reinforcing its occurrence. The consequence believed to reinforce the occurrence of the target's challenging behavior is removed, resulting in a decrease in the target behavior.
Functional Behavior Assessment (FBA)	It consists of a systematic way of determining the function or purpose of a behavior so that an effective intervention plan can be developed. FBA consists of describing the interfering or problematic behavior, identifying antecedent and consequent events that control the behavior (sometimes tested systematically through an Experimental Functional Analysis), developing a hypothesis of the function of the behavior, and testing the hypothesis, after which an effective intervention plan can be developed.
Functional Communication Training (FCT)	A set of practices that replace challenging behavior that serves a communication function with more appropriate and effective means of communication and behavioral skills.
Modeling (MD)	It involves demonstrating a desired target behavior that results in the student using the behavior and acquiring the target behavior. Thus, the student learns a specific skill through observational learning.
Music-mediated intervention	Interventions that incorporate songs, melodic intonation, and/or rhythm to support learning or performance of skills/behaviors. This included music therapy and other interventions that incorporate music to work on target behavior.
Naturalistic interventions (NI)	A collection of techniques and strategies incorporated into everyday activities and routines, in which students are naturally encouraged to develop target skills and behaviors. These practices are designed to encourage specific target behaviors based on students' interests, creating more complex skills that are naturally reinforcing and appropriate for interaction.
Parent-implemented intervention (PII)	Parents implement interventions with their children and promote their social communication skills, among other skills, while reducing challenging behaviors. The role of parents is to use the intervention practice to teach their children new skills, such as forms of communication, ways of playing, or self-care skills, involving their children in social communication and interactions, and/or how to reduce challenging behaviors.
Peer-based instruction and intervention (PBII)	Social interaction between peers is the defining characteristic of the intervention. Intervention in which peers directly promote the social relationships of children with autism, among other individual learning skills and goals. The adult organizes the social context (e.g., play groups and social contacts) and, when necessary, provides support (e.g., suggestions and reinforcement) to children with autism so that they can interact with their peers.
Tips (PP)	Verbal, gestural, or physical assistance provides students with the support they need to acquire or engage in target behavior.
Reinforcement (R)	It consists of applying consequences after the occurrence of a skill that increases the likelihood of compatible behaviors in future situations. Reinforcement includes positive reinforcement, negative reinforcement (different from punishment)
Response interruption/redirection (RIR)	It consists of introducing a hint, comment, or other distractor when undesirable behavior occurs, causing the student to shift their focus of attention, which results in a reduction in that undesirable behavior.
Self-management (SM)	It is an intervention package that teaches students to independently regulate their own behavior. Self-management involves teaching students to discriminate between appropriate and inappropriate behaviors, accurately monitor and record their own behaviors, and deliver reinforcers to themselves for behaving appropriately.
Sensory integration® (SI)	Interventions that aim to increase a person's ability to integrate sensory information (visual, auditory, tactile, proprioceptive, and vestibular) from the body and environment in order to respond using adaptive organizations and behavior.
Social Narratives (SN)	These are interventions that describe social situations in order to highlight relevant characteristics of a target behavior or skill and provide examples of appropriate behaviors. Social narratives aim to help students adapt to changes in routine, adapt their behaviors based on social and physical cues in a situation, or teach specific social skills or behaviors.
Social Skills Training (SST)	Individual or group instruction designed to teach students ways to participate appropriately and successfully in their social interactions.
Task Analysis (TA)	A process in which an activity or behavior is broken down into small, manageable steps to assess and teach the skill. Practices such as reinforcement, video modeling, or time delay are often used to facilitate the acquisition of even smaller steps.
Technology-assisted instruction and intervention (TAII)	Instruction or intervention in which the use of technology is the central feature and is designed and employed to support and enhance student learning or performance.
Time Delay (TD)	A practice used to systematically decrease the use of prompts during activity, using a brief delay between the instruction and any additional instruction or prompt. With progressive time delay, the practitioner gradually increases the waiting time between an instruction and any prompt that could be used to elicit a response from the learner. As the learner becomes more competent in using the skill, the practitioner gradually increases the waiting time between the instruction and the prompt.
Video Modeling (VM)	A method of instruction that uses video technology to record and present a demonstration of the desired behavior or skill. The demonstration is shown to the learner, who then has the opportunity to perform the target behavior at that time or at a later time.
Visual Supports (VS)	A visual aid that supports the student so that they can engage in desired behavior without additional help.

Despite the organizational and legal support for evidence-based practices, professionals and families of children with autism often turn to treatments lacking scientific validation. Several factors contribute to this. First, parents often experience a state of desperation after receiving their child's diagnosis, making them more willing to try anything at least once.^13^ Second, many parents experience overwhelming guilt, fearing they might one day regret not having tried “that one” treatment that could have worked for their child.^13^ Third, most parents have limited knowledge of autism, which remains a relatively uncommon condition, and even less familiarity with research-based interventions or the concept of rigorous scientific evaluation.^13^

Another important factor is the lack of consensus among professionals themselves. Unfortunately, evidence-based practices are still not the standard of care adopted by most professionals working in the autism field.^14^ Parents frequently receive conflicting opinions and recommendations, reflecting the limited number of professionals with solid training in evidence-based procedures. Without strong, consistent professional support for evidence-based practices, and instead receiving conflicting advice, families often feel confused and turn to a variety of sources for guidance, such as other families, the internet, and marketing materials. Finally, the very nature of ASD, with its highly individualized pattern of symptoms across a broad range of abilities, can create the impression that families must try many different approaches to find the “right” treatment for their unique child, reinforcing a sometimes endless and disorganized search for new options.

Although intensive behavioral interventions are often recommended for children with ASD, it is essential to recognize that simply increasing the number of weekly hours does not guarantee clinical effectiveness. Extensive interventions that are poorly structured or lack scientific grounding can consume time, resources, and energy without delivering meaningful progress. The central challenge, therefore, lies in balancing quantity and quality: ensuring that intensive programs are also individualized, supervised by qualified professionals, and grounded in robust evidence. Prioritizing quality means adopting clear, evidence-based practices, setting meaningful therapeutic goals, ensuring rigorous technical oversight, and actively engaging families, transforming the intensity of hours into a real investment in the child's development and quality of life.

## Practical recommendations for behavioral approaches in ASD: quality versus quantity

### Need for individualized treatment

After receiving an autism diagnosis, the next step for parents is to enroll their child in treatment. However, selecting the most appropriate intervention is not an easy task. Families of children with ASD gather information from a variety of sources, including personal anecdotes, parent groups, acquaintances, and especially the internet, which is often rich in misinformation. Frequently, the drive to seek alternative methods stems from dissatisfaction with or the apparent ineffectiveness of treatments previously adopted, contributing to the proliferation of approaches lacking proven efficacy.^15^

In recent years, there has been a notable expansion in available treatments for ASD. Parents and caregivers resort to various strategies, including pharmacological treatment, dietary changes or restrictions, and behavioral, educational, or alternative therapies. Notably, there is a tendency toward simultaneous use of multiple interventions and combinations of methods, often without clear coordination or rationale.^15^ However, it is essential to recognize that not all children progress at the same rate or to the same degree, making it imperative that each intervention plan be individualized and adapted to the specific needs of the child and family.^16^

Contrary to this principle of individualization, autism treatment often adopts a “one-size-fits-all” approach, offering interventions at predetermined intensities and frequencies without fully considering the child’s unique profile. Multidisciplinary collaboration, where professionals from diverse fields contribute their expertise in a coordinated effort, is essential to ensure sound planning, shared clinical judgment, and coherent decision-making in health, education, and social care settings. Such cooperation aims to provide a comprehensive understanding that combines knowledge across disciplines, transcending rigid professional boundaries.^16^

However, without a shared conceptual framework, multidisciplinary work risks devolving into disordered eclecticism characterized by uncoordinated and arbitrary choices of interventions. This can lead to undesirable side effects such as high costs, excessive workload, inefficiency, contradictions among approaches, professional competition, or even mutual nullification of treatment effects. Moreover, the lack of theoretical coherence and potential interpersonal conflicts fail to benefit service users, undermining the overall quality of care provided.^16^^,^^17^

In the most common models of interdisciplinary collaboration, there is often little consensus on the path to follow after diagnosis, with different professionals recommending interventions specific to their disciplines in an eclectic and frequently uncoordinated mix. In contrast, truly individualized, child-centered, evidence-based interventions must result from close collaboration among relevant professionals. Treatment decisions should be guided by carefully collected and analyzed behavioral data, ensuring an integrated approach that is responsive to the unique needs of each child.^17^

Every child with ASD presents a unique profile of strengths, challenges, preferences, and needs. Therefore, standardized “one-size-fits-all” models or fixed lists of interventions are inadequate. Conclusions from assessments and recommendations for treatment and support must always consider individual strengths, difficulties, and needs, as well as the family and social context in which the child lives. It is equally important to recognize that individuals, families, and circumstances evolve over time, requiring continuous adaptation of interventions and supports to maintain their relevance and effectiveness throughout development.^18^

**Figure 1:**
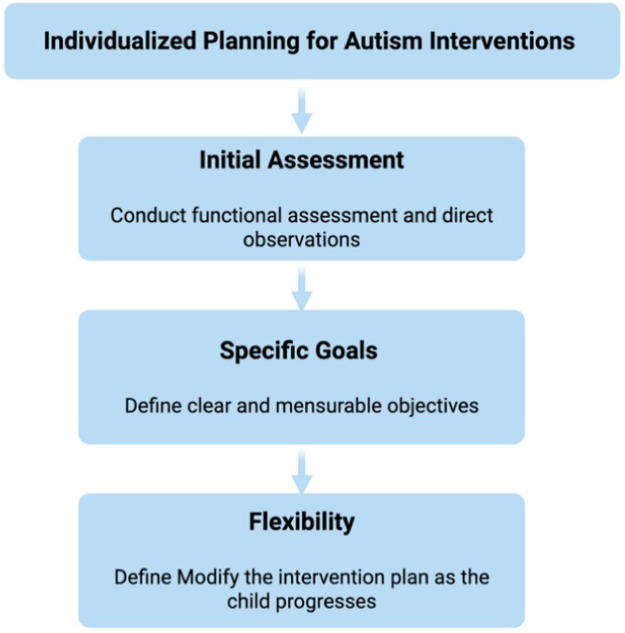
Individualized planning for autism interventions. A structured process outlining key steps for designing high-quality, individualized behavioral interventions for ASD.

To truly prioritize quality over simply increasing the quantity of hours or interventions indiscriminately, it is essential to conduct detailed functional assessments and direct observations to identify skills, deficits, target behaviors, and the family and social context. Such functional analysis should consider all factors that limit an individual’s capacity or quality of life, enabling the identification of underlying causes of difficulties, teaching more effective and acceptable alternative ways of interacting with the environment, and developing skills that improve quality of life. In addition, it is critical to establish clear, measurable goals aligned with the child’s and family’s priorities, and to maintain flexibility to adjust the intervention plan as the child evolves, adapting strategies to changes in development and behavior.^18^ Avoiding rigid, simultaneous application of multiple treatments without clear justification reduces the risk of overburdening the child and family and increases the effectiveness of interventions, reinforcing the principle that quantity has value only when accompanied by quality (Figure 1).

### Quality versus quantity: treatment response is not “dose-dependent”

A common misconception is that more hours of therapy automatically guarantee better outcomes. However, the quality of treatment is significantly more important than the sheer amount of time or number of therapy hours. A systematic review by Sandbank et al. analyzed effect sizes from quasi-experimental studies and randomized controlled trials, concluding that daily hours of therapy, number of days, and total hours were not systematically associated with better outcomes.^19^

**Figure 2:**
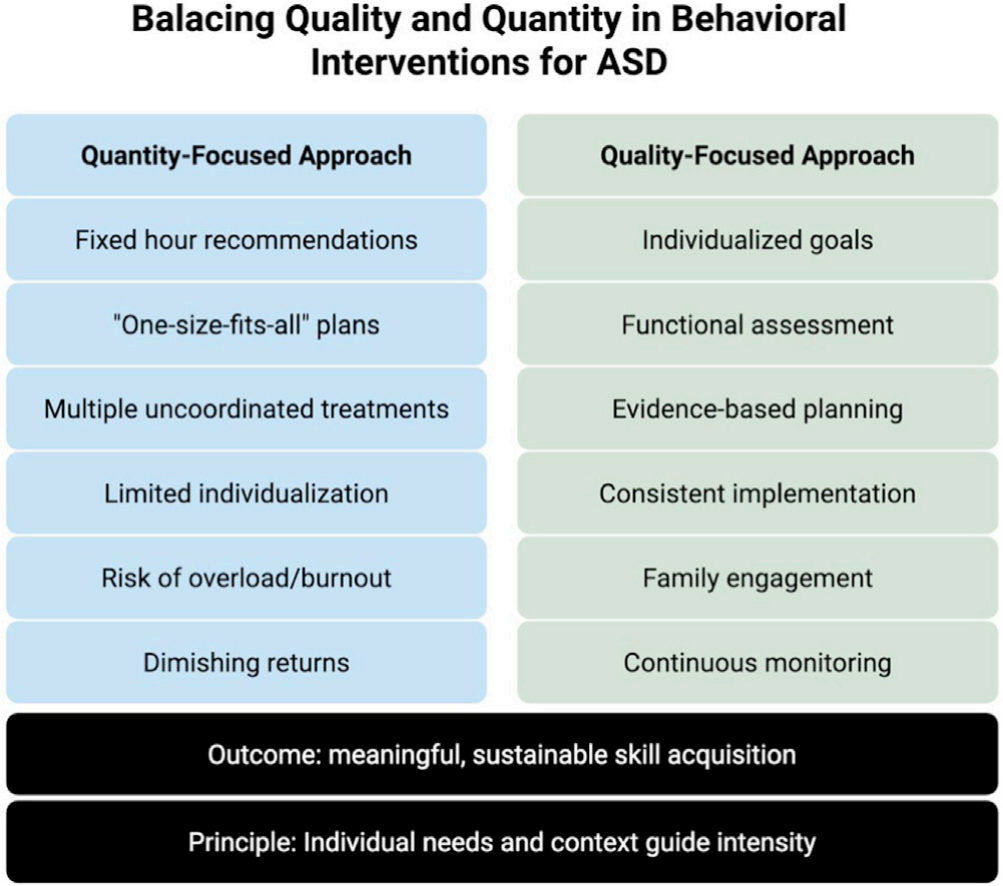
Balancing quality and quantity in behavioral interventions for ASD. A conceptual comparison illustrating risks associated with a quantity-focused approach (e.g., fixed hours, lack of individualization, diminishing returns) versus the benefits of a quality-focused approach emphasizing individualized goals, evidence-based planning, consistent implementation, family engagement, and continuous monitoring to achieve meaningful outcomes.

First, each person has a unique learning rate determined by various factors, including genetics, comorbid medical diagnoses (e.g., seizure disorders), and the quality of applied behavioral intervention. Second, there is an individual maximum for how much a person can learn, which varies widely: for some, the best achievable outcome may be living independently and maintaining employment; for others, it may be living in a group home with minimal staff support. Third, there are diminishing returns with additional hours of therapy, and excessive hours can even have negative impacts due to fatigue, overload, or reduced engagement (Figure 2).^20^

In other words, dose–response relationships are determined at the individual level, not the group level. The authors should not expect the same amount of progress from the same number of therapy hours for every individual. An illustrative example in ASD was published by Frazier et al., who demonstrated that the quantity of intervention can indeed be significantly associated with outcomes, but this relationship changes substantially when IQ is included in the analytic models.^21^

Therefore, rather than recommending a standardized number of hours for every autistic child, it is more important to ensure that each individual’s treatment includes evidence-based planning (using scientifically supported approaches such as Applied Behavior Analysis [ABA] or the Early Start Denver Model [ESDM]); consistent implementation with high fidelity to protocols tailored to the child's context; active family engagement, empowering parents and caregivers to reinforce skills at home and in other settings; and continuous monitoring, with regular assessment of progress using objective data to guide necessary adjustments.^19^, ^20^, ^21^

A high-quality intervention, even at lower intensity, can produce superior outcomes compared to many hours of poorly planned or poorly delivered therapy, underscoring the importance of prioritizing quality over quantity in any intervention strategy.

## Efficacy versus effectiveness: the role of therapist training

The two most important variables to ensure that individuals with autism acquire the skills needed to improve their overall quality of life and participate actively in society are (a) the instructional approach used and (b) the competence of the professionals delivering that instruction [22]. Until now, the authors have focused on the technical aspects of behavioral approaches applied to ASD treatment, reserving for this section the critical discussion of professional qualification.

When selecting a treatment, it is decisive to understand the difference between efficacy and effectiveness. Efficacy refers to whether an intervention produces a specific outcome under controlled or “optimal” conditions, typically involving a highly selected population receiving treatment from well-trained clinicians in ideal settings.^23^ Effectiveness, on the other hand, measures whether an intervention achieves specific outcomes in “real-world” clinical conditions, where samples are more heterogeneous and factors such as the setting, family adherence to treatment, and therapist competence directly influence success.^23^

Using Applied Behavior Analysis (ABA) as an example, ensuring that professionals with solid training in behavior analysis are equipped to work with individuals with ASD is a critical concern. Unfortunately, the number of trained professionals remains far from meeting the demand. Moreover, many families of children with ASD lack clear information and standards for what constitutes adequate training in ABA, making it difficult to identify qualified practitioners. This situation creates opportunities for individuals without the necessary training or experience to continue offering services.^22^

To address this need in the United States, the Behavior Analyst Certification Board (BACB) was established. Professionals can become certified at two levels: Board Certified Behavior Analyst (BCBA) and Board Certified Assistant Behavior Analyst (BCaBA). Each level has specific requirements for academic degrees, coursework hours, and practical experience to qualify for the written exam. BCBAs must hold at least a master's degree and complete 180 instructional hours in graduate-level behavior analysis coursework, equivalent to 12 semester credit hours or four advanced courses. Approximately two-thirds of these hours must cover basic principles of behavior and their application, single-subject design, and ethics, with the remaining third chosen by the candidate. Additionally, candidates must complete at least 18 months of supervised experience in an appropriate applied setting. BCaBAs must hold at least a bachelor's degree and complete 90 instructional hours (6 semester credits) in the same core areas, along with at least 9 months of supervised experience. Both levels require passing a written exam, and to maintain certification, practitioners must complete specific continuing education units, ensuring they stay current with developments in behavior analysis and professional practice.^22^

In Brazil, professionals offering ABA services are often called “behavior analysts” regardless of their level of training. However, the profession of behavior analyst is not formally recognized in Brazil, and there is no regulation defining minimum training requirements, quality standards, or any restrictions on offering ABA services. This lack of regulation underscores the critical need to discuss the professional qualifications of those providing care to individuals with ASD.^24^

## Conclusion

Ensuring high-quality behavioral interventions for individuals with ASD requires not only solid scientific evidence but also well-trained professionals, individualized planning, and thoughtful interdisciplinary collaboration-underscoring that more hours or uncoordinated approaches cannot replace a genuine commitment to meaningful outcomes and respect for each individual’s unique needs.

## Funding support

No funding support was received for the completion of this work.

## Conflicts of interest

The authors declare that they have no conflicts of interest. All authors read and approved the final manuscript.
